# Fortifying our commitment to pediatric academic medicine during turbulent times

**DOI:** 10.1038/s41390-025-04621-w

**Published:** 2025-11-26

**Authors:** Mona Patel Gera, Arvin Garg, Maya I. Ragavan

**Affiliations:** 1 Department of Pediatrics, University of Southern California, Los Angeles, CA USA; 2 Department of Pediatrics, Children’s Hospital Los Angeles, Los Angeles, CA USA; 3 Department of Pediatrics, Child Health Equity Center, Worcester, MA USA; 4 University of Pittsburgh, Pittsburgh, PA USA

“There can be no keener revelation of a society’s soul than the way in which it treats its children.” — Nelson Mandela, May 8, 1995

Academic Medicine, with its tripartite mission to advance medical science, cultivate the next generation of healthcare professionals, and provide exceptional clinical care, offers unparalleled opportunities to shape the future of evidence-based healthcare delivery and inform health policy. Biomedical innovation developed through collaboration between academic medicine and public health can improve health at the individual and population health levels including mapping disease trends and improving treatment outcomes.^1^ According to the Association of American Medical Colleges (AAMC) economic impact report published in 2022, every one dollar spent by the AAMC member medical schools and teaching hospitals contributes $1.62 to the United States economy.^2^ Unfortunately, medical schools and teaching hospitals continue to face reductions in government funding for research and education support and have faced decades of under-reimbursement for publicly insured care, often delivered in these systems which also serve as safety net health care systems across the country.

The National Institutes of Health (NIH) revealed that the nation’s supply of physicians engaging in research continues to decline, with increased competition for decreasing available federal funding.^3^ Pediatric research is disproportionately impacted by gaps in funding, directly impacting the scientific innovation aimed at improving child health outcomes.^4,5^ As advanced technology such as artificial intelligence (AI) balanced with rapidly evolving scientific discoveries offer novel and dynamic clinical approaches to care, these persistent funding gaps often lead to pediatric care falling behind adult care delivery. With an estimated 646,000 researchers supported by federal grants, 48% of whom are students and trainees, the cross-cutting dependence and impact of federal funding on research and education is undeniable.^6^ Therefore, to mitigate further impact in pediatric academic medicine, continued promotion of NIH-funded pediatric research must leverage the understanding that return on the investment continues over the lifespan of the growing child to adult.^7^

In the article by Arnaez and colleagues, Dr. Garcia-Alix is celebrated for his longitudinal contributions and global reach in academic medicine, specifically in field of Neonatal Neurology. By understanding that at least 30% of neonates admitted to Neonatal Intensive Care Units (NICUs) present with neurological pathology or other conditions impacting brain development, he recognized the importance of studying neuroprotective factors that impact these children’s morbidity. For Dr. Garcia-Alix, this area of interest sparked a career long passion. His dedication was evident, while advancing and supporting academic education and mentorship of his learners, generously investing time and role modeling for further generations. With the appreciation of the life-course model for his neuro-neonatal patients, Dr. Garcia-Alix, translated the overarching goal of improvement of functional outcomes by creating Brain-Aware Care, a family-centered, multidisciplinary approach targeting protecting an infant’s developing brain at the earliest stages. He recognized and appreciated that neuro-neonatal care delivery differs requires a comprehensive, multidisciplinary, family-centered approach tracking continuity of care as a baby grows, as is the continued research to understand how to develop appropriate care guidelines and protocols for developing, growing and thriving children. Dr. Garcia-Alix dedication and contributions as a physician scientist and educator with global impact on generations of patients in the field Neonatal Neurology is a testament of the longitudinal investment and support required to achieve the return on investment in child health outcomes in pediatric academic medicine.

## Fiscal pressure on the future of pediatric academic medicine

The goal of pediatric academic medicine is to continue to develop and support clinician scientists like Dr. Garcia-Alix throughout their careers, who are committed to innovation, clinical care, and education. However, the fiscal pressure on pediatric academic medicine for the past several years, balancing under-reimbursed Medicaid clinical activity, rising labor costs, inadequate availability of pediatric extramural funding, and insufficient graduate medical education funding, is leaving teaching hospitals and partnered medical schools to find novel ways to advance this pediatric academic mission. Institutional-level investment in clinician scientists (e.g., 3 year contracts where early career faculty have time to successfully compete for a career development grant) are less feasible as financial pressure mount from funds flow models in Children’s Hospitals.^7,8^ Similarly, competition for institutional (e.g., KL2) and external (e.g., K23) grants are increasing as paylines are decreasing. With recent changes in administration policy, opportunities for first-generation scientists and those underrepresented in biomedical research (e.g., diversity supplements, MOSAIC award) are now unavailable. Protecting NIH funding, particularly money appropriated to child health research, is essential to promote the mission of pediatric academic medicine. Moreover, given diminishing availability of federal extramural funding, reviewing alternative, non-traditional academic sources of funding mechanisms for research including private industry, venture capital or foundation funding will be necessary to explore to uphold the tripartite mission of our pediatric academic mission.

In addition to research challenges, pediatric residency training programs are in jeopardy. Since the annual budget for graduate medical education is supported through the Congressional appropriations processes, strategic consideration of alternative platforms of support for retention, learner mentorship, and workforce pipelines are critical to ensure continued access to care for our pediatric population. Mentors can be instrumental at different stages of learners, from guiding students in explicit academic knowledge, to implicit knowledge of professionalism, ethics and the art of medicine.^9^ Retention of faculty is critical during times of economic stressors in academic medicine and leaning on mentorship and sponsorship as a key strategy, especially given pay inequity between pediatric and adult academic clinicians. Finally, evaluation of the economics of health professions education (HPE), is critical with ongoing cost-constrained academic medicine environments, especially with graduate medical education funding at risk.^10^

## Call to action for the health of our nation’s children

We need to reinforce and double-down on our commitment to the pediatric academic medicine mission-centered goal in improving the health and well-being our children and youth through investments in advancing science, cultivating our next generation of pediatric learners and improving clinical care for our pediatric patients. With several decades of Medicaid under-reimbursement, challenging degradation of federal pediatric extramural funding compared with adult funding, and even greater disparities in pediatric workforce shortages, families are facing a stark reality in worsening access to pediatric healthcare which will only grow in the upcoming years due to Medicaid cuts. We must continue to educate legislators and government officials that children require the same level of extramural funding (*if not greater*), in efforts to evolve even greater investment on return investment over the life-course trajectory. We must advocate for reimbursement parity greater than Medicare, especially understanding the discrepancy between Medicaid enrollment and Medicaid expenditure on children, being roughly 50% and 20%, respectively. Finally, we must educate that pediatric academic medicine, with its medical schools and teaching hospitals, contribute to the economy and health of the United States.

## Call to action

- Support the Bipartisan Legislation H.R. 3890 (Sewell, Fitpatrick)- Resident Physician Shortage Reduction Act of 2025- Educate your local legislators on the importance of Pediatric Education workforce pipeline and its’ impact on access to care, especially in rural communities
- Continuing to unequivocally advocate for Children’s Hospitals Graduate Medical Education (CHGME), and budget appropriation within the annual Labor Health and Human Services Appropriations Process for the House and Senate Committees
- Support NIH funding increases for Pediatric Research funding, which will increase and parity (and increase) for pediatric extramural funding for research to improve the evidence-based decision-making, and uphold trust with patients, families and society
- Continue to educate our legislators about the life-course model in child health, and the return on investment, with the reality that for every $1 investing in early intervention, there is a return on investment from $1.26 to $17.07 for every child invested in the United States.^11^
- Finally, ensure that with the rapid advancing in technology, including artificial intelligence (AI), gaps do not arise regarding access to therapeutic tools between pediatric and adult populations in healthcare.^12^
